# Involvement of Opioid System, TRPM8, and ASIC Receptors in Antinociceptive Effect of *Arrabidaea brachypoda* (DC) Bureau

**DOI:** 10.3390/ijms18112304

**Published:** 2017-11-02

**Authors:** Vinícius Peixoto Rodrigues, Cláudia Quintino da Rocha, Larissa Lucena Périco, Raquel de Cássia dos Santos, Rie Ohara, Catarine Massucato Nishijima, Emerson Ferreira Queiroz, Jean-Luc Wolfender, Lúcia Regina Machado da Rocha, Adair Roberto Soares Santos, Wagner Vilegas, Clélia Akiko Hiruma-Lima

**Affiliations:** 1 São Paulo State University (UNESP), Biosciences Institute, Department of Physiology, Botucatu 18618-970, SP, Brazil; viniciuspr42@gmail.com (V.P.R.); larissalucenaperico@gmail.com (L.L.P.); rieohara@gmail.com (R.O.); catarinenishijima@gmail.com (C.M.N.); lrocha@ibb.unesp.br (L.R.M.d.R.); 2 Federal University of Maranhão, Department of Chemistry, Av. dos Portugueses, 1966—Bacanga, São Luís 65080-805, MA, Brazil; claudiarocha3@yahoo.com.br; 3 Clinical Pharmacology and Gastroenterology Unit (USF), Bragança Paulista 12916-900, SP, Brazil; raquel.cassia@usf.edu.br; 4 School of Pharmaceutical Sciences, EPGL, University of Geneva, University of Lausanne, CMU–Rue Michel-Servet 1, CH-1211 Geneva 4, Switzerland; emerson.ferreira@unige.ch (E.F.Q.); Jean-Luc.Wolfender@unige.ch (J.-L.W.); 5 Federal University of Santa Catarina, Laboratory of Neurobiology of Pain and Inflammation, Department of Physiological Sciences, Center of Biological Sciences, Trindade, Florianopolis 88040-900, SC, Brazil; adair.santos@ccb.ufsc.br; 6 São Paulo State University (UNESP), Biosciences Institute, São Vicente 11330-900, SP, Brazil; vilegas@gmail.com

**Keywords:** *Arrabidaea brachypoda* (DC) Bureau, Bignoniaceae, antinociceptive effect, pain

## Abstract

*Arrabidaea brachypoda* (DC) Bureau is a medicinal plant found in Brazil. Known as “cipó-una”, it is popularly used as a natural therapeutic agent against pain and inflammation. This study evaluated the chemical composition and antinociceptive activity of the dichloromethane fraction from the roots of *A. brachypoda* (DEAB) and its mechanism of action. The chemical composition was characterized by high-performance liquid chromatography, and this fraction is composed only of dimeric flavonoids. The antinociceptive effect was evaluated in formalin and hot plate tests after oral administration (10–100 mg/kg) in male Swiss mice. We also investigated the involvement of TRPV1 (transient receptor potential vanilloid 1), TRPA1 (transient receptor potential ankyrin 1), TRPM8 (transient receptor potential melastatin 8), and ASIC (acid-sensing ion channel), as well as the opioidergic, glutamatergic, and supraspinal pathways. Moreover, the nociceptive response was reduced (30 mg/kg) in the early and late phase of the formalin test. DEAB activity appears to involve the opioid system, TRPM8, and ASIC receptors, clearly showing that the DEAB alleviates acute pain in mice and suggesting the involvement of the TRPM8 and ASIC receptors and the opioid system in acute pain relief.

## 1. Introduction

Historically, the study of plants used for medicinal purposes accompanied and promoted the discoveries and development of pure drug substances and, consequently, new therapeutics. Although medicinal plants have been explored for an extensive period, they still present themselves as a remarkable source of new bioactive substances with therapeutic potential [1,2].

Brazilian flora is distributed in extremely diverse ecosystems. Contributing to this diversity, the Cerrado (neotropical savanna) extends mainly over the northeast, middle west, and southeast of Brazil and contains more than 12,000 plant species [3].

*Arrabidaea brachypoda* (DC) Bureau is a medicinal plant of the Brazilian Cerrado ecosystem, but few pharmacological and phytochemical studies related to this species can be found in literature. Its roots are used in traditional medicine for the treatment of joint pain [4]. Furthermore, we have demonstrated that the crude hydroethanolic extract of the roots—the composition of which is closely related to the traditional decoction—showed gastroprotective and gastrointestinal healing properties, as well as anti-inflammatory and antinociceptive activities in animal models [5,6]. But the compound(s) responsible for this antinociceptive effect, or even the underlying mechanisms of action whereby this medicinal plant reduces pain, are not well understood.

An extensive phytochemical study of this extract revealed the presence of the glycosylated forms of dimeric flavonoids [5]. Our previous study with the dichloromethane (DCM) fraction of the roots revealed that these three unusual dimeric flavonoids isolated from this fraction present high in vitro and in vivo activities against *Trypanosoma cruzi*, the parasite responsible for the Chagas disease [7].

In this study, we propose to investigate the antinociceptive effect of this dichloromethane fraction of *A. brachypoda*, which has mainly unusual dimeric flavonoids in phytochemical compounds. Furthermore, this study will determine the antinociceptive mechanism(s) of action of this medicinal plant.

## 2. Results and Discussion

**Figure 1:**
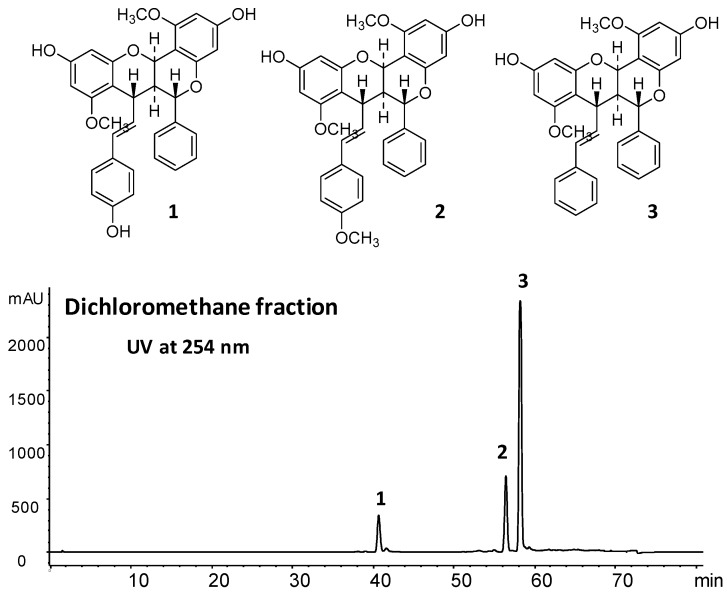
HPLC with photodiode array detection (HPLC–PDA) chromatogram of dichloromethane fraction of samples obtained from the roots of *Arrabidaea brachypoda.* This chromatogram presents three dimeric flavonoids named brachydin A (**1**); brachydin B (**2**); and brachydin C (**3**).

Previous studies have already demonstrated antinociceptive activity by a hydroethanolic extract from the roots of *A. brachypoda* in rodents, but the underlying mechanisms of pain reduction are not well understood [5]. In the present study, we extend previous data using a DCM (dichloromethane) fraction extracted from the hydroethanolic root extract. The dichloromethane fraction of *A. brachypoda* was obtained by a liquid/liquid partition from the hydroethanolic root extract as described in our previous study [7]. The HPLC–PDA (high-performance liquid chromatography with photodiode array detection) analysis revealed only three major compounds that were identified by comparison with pure isolated standards (Figure 1). These compounds were previously fully characterized as three unusual dimeric flavonoids named brachydin A (1), brachydin B (2), and brachydin C (3) [7].

**Figure 2:**
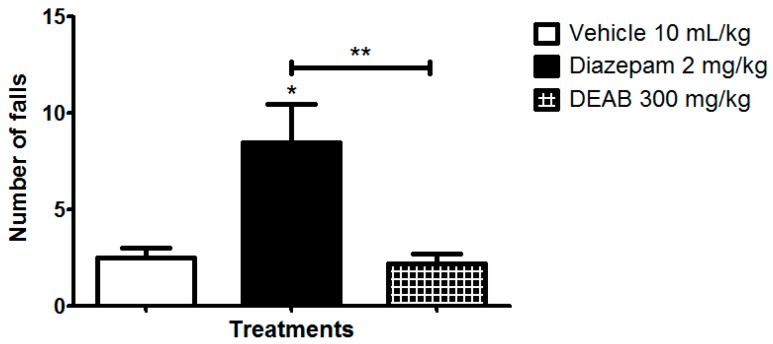
Effect of the dichloromethane fraction of samples obtained from the roots of *Arrabidaea brachypoda* (DEAB) on the locomotive performance of mice in a rotarod test. The results are the number of falls in each group of animals expressed as the mean of the values obtained in 8 animals ± S.E.M. One-way ANOVA (analysis of variance) followed by Dunnett’s test (all vs. vehicle) and Student’s *t*-test (Diazepam vs. DEAB); * *p* < 0.05, ** *p* < 0.01.

There is evidence that myorelaxant drugs or sedatives can promote changes in locomotor performance in mice, resulting in false positive results for drugs with antinociceptive effects [8,9]. Thus, to exclude this possibility, we evaluated the integrity of locomotor performance of the mice treated with DEAB on a rotarod apparatus. Pretreatment with DEAB did not significantly affect the locomotor performance of the animals after oral administration of 300 mg/kg (the maximum dose used in this study). Only the positive control (diazepam, 2 mg/kg) group (*p* < 0.01) significantly decreased the endurance time on the rotating rod when compared with the time of the control group (Figure 2). Therefore, after ensuring that the DEAB would not cause muscle relaxation or sedation when acutely administered, the antinociceptive effect could then be tested.

The formalin test is a widely used model of persistent nociception and is a mainstay for the development of novel agents for the treatment of acute and chronic pain [9,10]. The test shows a biphasic response. The first phase (inhibited by narcotic drugs such as morphine) begins immediately after the formalin injection and is caused by direct action of the solution on the local sensory C-fibers, resulting in the release of calcitonin gene-related peptide (CGRP) and substance P [11,12]. The second phase (15–30 min after injection) is associated with inflammatory pain due to the release of inflammatory mediators, such as prostaglandins and nitric oxide, and is responsive to non-steroidal anti-inflammatory drugs (NSAIDs) [10,11,13,14].

**Figure 3:**
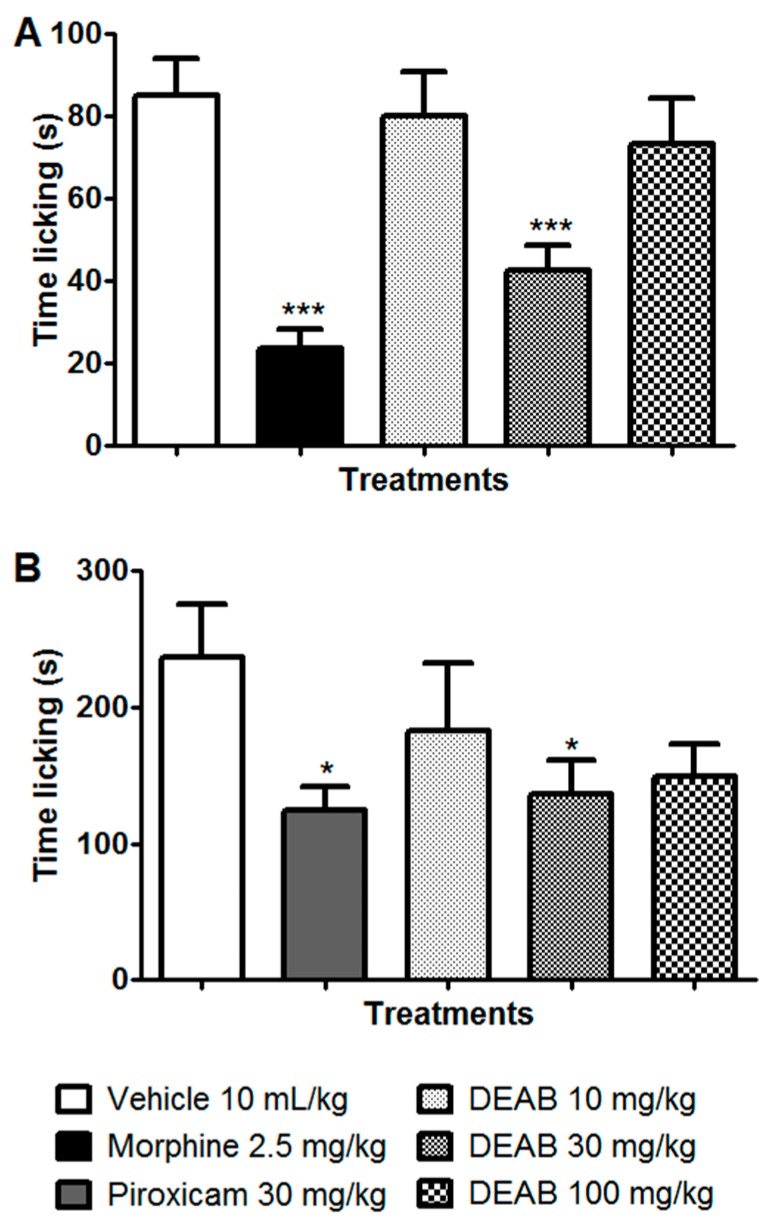
Effect of DEAB (10–100 mg/kg, oral), morphine (2.5 mg/kg, subcutaneous), or piroxicam (30 mg/kg, oral) on nocifensive behavior induced by intraplantar injection of formalin in mice. The total time spent licking the hind paw was measured during the (**A**) neurogenic phase (0–5 min) and the (**B**) inflammatory phase (15–30 min). The results are expressed as mean of the values obtained in 8–10 animals ± S.E.M. One-way ANOVA followed by Dunnett’s test (all vs. vehicle); * *p* < 0.05, *** *p* < 0.001. Student’s *t*-test (Piroxicam vs. DEAB 100 mg/kg or DEAB 30 mg/kg vs. DEAB 100 mg/kg).

The results depicted in Figure 3 show that the intraplantar injection of formalin resulted in a typical biphasic nocifensive behavior. Mice spent approximately 90 and 250 s displaying nociceptive behaviors during the first 5 min (neurogenic pain, phase 1) and the subsequent 15–30 min (inflammatory pain, phase 2) of the assay, respectively (Figure 3A,B). In the neurogenic phase, morphine (2.5 mg/kg) and DEAB (30 mg/kg) reduced nociceptive behavior by 72% (*p* < 0.001) and 50.1% (*p* < 0.001), respectively, when compared with that of the vehicle-treated group (Figure 3A). During the inflammatory phase, the positive control (piroxicam, 30 mg/kg) and DEAB at 30 mg/kg reduced hind-paw licking by 47.2% (*p* < 0.05) and 42.7% (*p* < 0.05), respectively, compared with that of the negative control (Figure 3B). These results show that, in contrast to the crude polar extract of *A. brachypoda* that shows antinociceptive activity only during the second phase (inflammatory pain), the DCM fraction can induce antinociceptive effects during both phases. Although the superior dose of DEAB (100 mg/kg) was not able to significantly reduce the nociceptive behavior caused by formalin when compared with the vehicle-treated group (Figure 3B), there is a decrease response of DEAB (37%) in relation to the control group. This reduced nociceptive behavior is also evident when we compared the DEAB-treated group (100 mg/kg) with the piroxicam-treated group, or the DEAB-treated group (100 mg/kg) with the DEAB-treated group (30 mg/kg), in which there is no significant difference between these groups (Student’s *t*-test, *p* > 0.05). It is likely that the superior dose of DEAB used may have been very high, and needed more time to exert their antinociceptive effect; this would explain this reduction in the inflammatory phase evaluated 30 min after the administration of DEAB and not in the first phase. Hence, the subsequent experiments with DEAB were carried out at the effective dose of 30 mg/kg.

**Figure 4:**
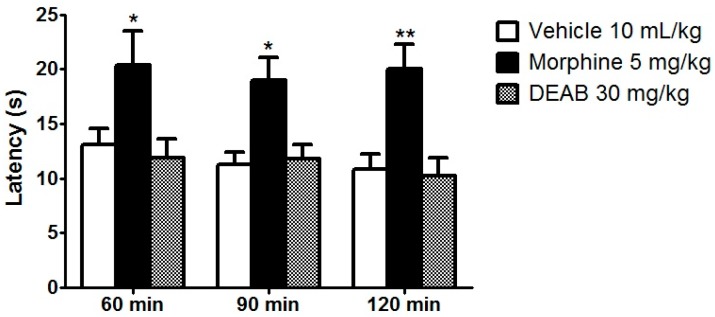
Effects of the dichloromethane fraction of samples obtained from *Arrabidaea brachypoda* root (DEAB) or morphine (5 mg/kg) on the supraspinal reflexes of mice in the hot plate test. The results are the time to exhibit a nociceptive response to a hot plate expressed as the mean of the values obtained in 8 animals ± S.E.M. Two-way ANOVA with a Bonferroni correction for multiple comparisons (all vs. vehicle); * *p* < 0.05, ** *p* < 0.01.

After verifying the antinociceptive activity of the DEAB, its mechanisms of action were investigated. The hot plate test is an easy and reliable method that is capable of evoking supraspinal responses [15]. However, DEAB showed no antinociceptive activity against supraspinal responses. The DEAB-treated group showed the same latency response as the vehicle-treated group did (*p* > 0.05) throughout the observation period (Figure 4).

Noxious thermal, mechanical, or chemical stimuli evoke pain through excitation of the peripheral terminals called nociceptors. Many kinds of ionotropic and metabotropic receptors are involved in this process, such as transient receptor potential (TRP) and acid-sensing ion channels (ASIC) [16]. The TRP channels are divided into seven subfamilies, and four of them are involved with nociception [17]. TRPV1 is sensitive to capsaicin and noxious heat, TRPA1 is activated by a wide variety of chemical agents such as isothiocyanates and cinnamaldehyde, and TRPM8 responds to noxious cold as well as menthol [18,19]. TRPV1, TRPM8 and TRPA1 are greatly implicated in the pathogenesis of acute and chronic pain and, according to Stucky et al. [20], these channels are the main TRP ion channel family that detect noxious stimuli and transduce a diverse range of physical and chemical energy into action potentials in somatosensory nociceptors. ASICs are cationic channels from the degenerin/epithelial sodium channel (DEG/ENaC) family, which are involved in the nociception to elevated extracellular H^+^ concentrations in both the central and peripheral nervous systems [21].

**Figure 5:**
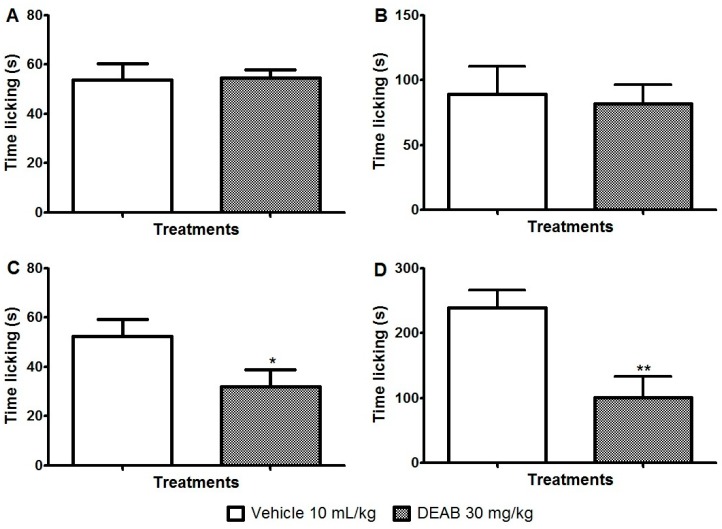
Effects of the dichloromethane fraction of samples obtained from *Arrabidaea brachypoda* root (DEAB) on the nocifensive behavior of mice induced by an intraplantar injection of (**A**) capsaicin (2 µmol/paw); (**B**) cinnamaldehyde (40 nmol/paw); (**C**) menthol (2 μmol/paw); and (**D**) acidified saline (pH 2.0/paw). The results are the time the animals spent licking their right hindpaw expressed as the mean of the values obtained in 8 animals ± S.E.M. Student’s *t*-test; * *p* < 0.05, ** *p* < 0.01.

**Figure 6:**
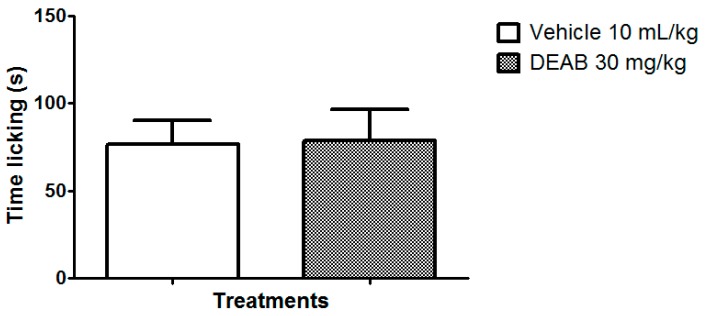
Effects of the dichloromethane fraction of samples obtained from *Arrabidaea brachypoda* root (DEAB) on the nocifensive behavior of mice induced by an intraplantar injection of glutamate (30 µmol/paw). The results are the time the animals spent licking their right hind paw expressed as the mean of the values obtained in 8 animals ± S.E.M. Student’s *t*-test; *p* > 0.05.

The involvement of these channels in the antinociceptive mechanism of DEAB was evaluated. Our results showed that DEAB, at the dose that was effective in reducing inflammatory and neurogenic pain in the formalin test, did not reverse the nociception caused by capsaicin (an activator of the TRPV1 channel) or cinnamaldehyde (an activator of the TRPA1 channel) when compared with the control group (*p* > 0.05) (Figure 5A,B). DEAB was also ineffective against the nociceptive behavior caused by glutamate (*p* > 0.05) (Figure 6). Interestingly, DEAB significantly reduced the nociceptive behavior induced by menthol (an activator of the TRPM8 channel, 38.7%, *p* < 0.05) and acidified saline (an activator of the ASIC channel, 57.9%, *p* < 0.01) when compared with the vehicle-treated group (Figure 5 C,D).

These results confirm the data in which DEAB decreased the nociception induced by formalin in both phases, since ASIC receptors are activated by extracellular acidosis, which is caused by tissue damage and inflammation [22]. Proton-sensing receptors can be divided into ion channels (TRPV1 and ASIC family) and G-protein-coupled receptors (GPCR) [23,24]. There is evidence that proton-sensing GPCR are involved with the modulation of inflammatory pain acting on TRPV1 and also the ASIC family [25,26,27]. Since DEAB showed no effect over TRPV1 and exhibited a diminished response in the acidified saline model, combined with its anti-inflammatory effect already observed by Da Rocha et al. [5], we cannot rule out the hypothesis of the direct or indirect action of the extract though the inhibition of GPCRs.

TRPM8 is a major cold and cooling transduction channel in mammalian sensory neurons and it is strongly activated by the cool-mimetic chemical menthol and by physical cooling [20]. This channel is the principal mediator of menthol-induced analgesia of acute and inflammatory pain [28]. Andersson et al. [29] described that the inhibition of phospholipase A2 (PLA2) can prevent TRPM8 activation by cold, ilicin, and menthol. Therefore, this observation would likely explain the antinociceptive effect of DEAB observed in the second phase of the formalin test.

The opioid system plays an important role in the transmission and modulation of noxious stimuli. This occurs via peptides and receptors expressed throughout the nervous system [30]. The opioid system acts by modulating the ion channels of the neurons in the descending pathways of nociception. When activated, the opioid receptors inhibit the influx of Ca^2+^ in the presynaptic fibers, reducing the release of neurotransmitters, while hyperpolarizing the postsynaptic fibers due to K^+^ efflux [30].

**Figure 7:**
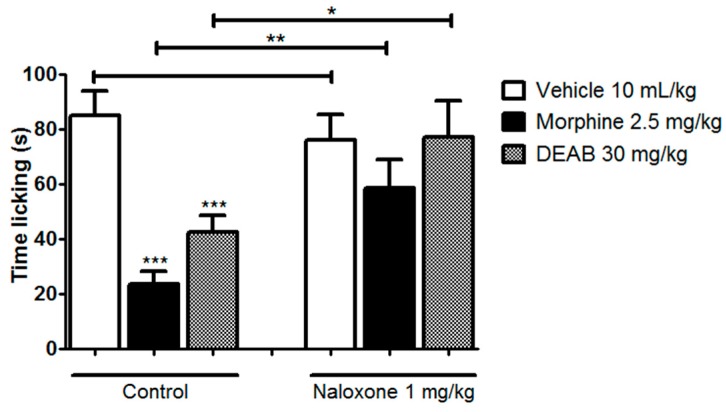
Involvement of opioid system in antinociceptive effect of the dichloromethane fraction of samples obtained from *Arrabidaea brachypoda* root (DEAB) in mice previously treated with saline (10 mL/kg, intraperitoneal: i.p.) or naloxone (1 mg/kg, i.p., a nonselective opioid receptor antagonist) against nocifensive behavior induced by intraplantar injection of formalin in mice. The total time spent licking the hind paw was measured during the neurogenic phase (Early phase, 0–5 min). The results are expressed as mean of the values obtained in 8–10 animals ± S.E.M. One-way ANOVA followed by Dunnett’s test (all vs. vehicle within pre-treatment groups) and Student’s *t*-test (similar treatments of different pre-treatment groups); * *p* < 0.05, ** *p* < 0.01 and *** *p* < 0.001.

Although the hot plate test is a method that is related to evoking supraspinal responses, this method is also known as the test able to certify the involvement of central opioid receptors in the mechanism of pain suppression [31]. Our result in Figure 4 initially indicates that DEAB does not present antinociceptive activity against supraspinal responses and also indicates non-involvement with opioid receptors. However, Endoh et al. [32] have already shown that κ-opioid receptor agonists are ineffective in inhibiting the hot plate response at 55 °C and other tests that employ heat as the nociceptive stimulus. The hot plate test is selective for µ-agonists and insensitive to the antinociceptive actions of κ-agonists [31]. To evaluate this hypothesis—whether the opioid system contributes or not to the antinociceptive effect of DEAB—we performed a formalin test with a naloxone (a non-selective antagonist of opioid receptors) pre-treatment. Based on the results shown in Figure 7, we have confirmed the above findings that DEAB inhibits the nociceptive response induced by intraplantar injection of formalin, and have also shown that naloxone completely reverses the antinociception caused by morphine (*p* < 0.01) and DEAB (*p* < 0.05), confirming that the opioid system also contributes to the analgesic effect of the DEAB. Considering the results obtained with this study, we can affirm that the studies with DEAB warrant a continuation. It is still necessary to further investigate the peripheral and central effect of the extract in the opioid receptor subtypes. Another point that requires further attention is the relationship of the extract with the opioid system in inflammatory pain modulation. Moreover, given all the evidence, the specificity of DEAB—particularly in relation to κ receptors—needs to be confirmed. Thus, the continuity of the studies will provide a better understanding of the mechanisms underlying DEAB effects.

## 3. Materials and Methods

### 3.1. Chemicals and Reagents

The chemicals that were used are as follows: acetic acid (Labimpex, Diadema, Brazil), formaldehyde (Chemco, Campinas, Brazil), capsaicin, cinnamaldehyde, menthol, l-glutamic acid hydrochloride (Sigma-Aldrich, St. Louis, MO, USA), naloxone (Tocris Cookson Ltd., Bristol, UK), morphine (Cristália, Itapira, Brazil), diazepam (Hipolabor, Belo Horizonte, Brazil), piroxicam (Pfizer, São Paulo, Brazil), methanol (MeOH, Sigma-Aldrich, HPLC grade), dichloromethane (DCM, Tedia, Rio de Janeiro, Brazil), and formic acid (Tedia, Rio de Janeiro, Brazil). A saline solution (0.9% NaCl) was used as the vehicle for the drugs, and the solutions were adjusted to pH 7.0 with 3 M NaOH, if necessary.

### 3.2. Collection and Identification of Plant Samples

Samples of *A. brachypoda* roots were collected in April 2013 at the Sant’Ana da Serra farm in João Pinheiro, Minas Gerais, Brazil (Location: 17°44′45′′ S, 46°10′44′′ W). The plant was identified at the Instituto de Ciências Exatas e Biológicas (ICEB) by Maria Cristina Teixeira Braga Messias from the José Badine Herbarium of the Federal University of Ouro Preto. A voucher specimen (No. 17935) was deposited at the Herbarium of the Federal University of Ouro Preto, Minas Gerais, Brazil.

### 3.3. Preparation of the Fraction and Isolation

The dried roots (300 g) were successively extracted by percolation, at room temperature, with 70% ethanol. The crude hydroethanolic extracts were filtered and evaporated to dryness under vacuum at approximately 40 °C, yielding 11.8 g of dried hydroethanolic extract. The hydroalcoholic extract was partitioned with DCM and a methanol–water (7:3) mixture. The crude DCM fraction was obtained after decantation and evaporation to dryness under vacuum at approximately 40 °C, yielding 37.7% (4.44 g) based on the dry mass. Each fraction was analyzed further by HPLC–PDA using the followed conditions: HPLC–PDA data were obtained with an Agilent HP 1100 series system consisting of an autosampler, high-pressure mixing pump, and PDA detector (Agilent Technologies, Santa Clara, CA, USA). The HPLC conditions were as follows: an XBridge C-18 column (5 µm, 250 × 4.6 mm internal diameter) (Waters, Middleton, WI, USA); solvent system: A, MeOH containing 0.002% formic acid and B, H_2_O containing 0.002% formic acid; gradient: 5% to 100% of A in 60 min followed by 100% of A for 10 min; flow rate: 1 mL/min; injection volume: 10 µL; and sample concentration: 10 mg/mL in MeOH. The UV absorbance was measured at 210 and 254 nm, and UV spectra (PDA) were recorded between 190 and 600 nm (in increments of 2 nm). The identification of the compounds was performed by the comparison of the retention time with isolated standards of compounds **1**, **2** and **3**.

### 3.4. Animals

Adult male Swiss mice (20–35 g) were obtained from the Anilab Laboratory Animal Creation and Trade Ltd. (Paulínia, São Paulo, Brazil). All animals were housed collectively in cages and were kept in a controlled environment (22 ± 2 °C, with a 12 h light/dark cycle, lights on at 06:00) with access to water and food (Presence^®^, Paulínia, Brazil) ad libitum. Animals were allowed to acclimatize to housing conditions for at least seven days before experiments, and all experiments were performed during the light phase of the light/dark cycle. All experiments conducted were in accordance with the Brazilian legislation regulated by the National Council for the Control of Animal Experimentation (CONCEA) and ethical principles in animal research formulated by the Brazilian Society of Science in Laboratory Animals. The animal protocol was approved by the Biosciences Institute/UNESP Ethics Committee on Use of Animals (Approval No.: 728-CEUA; Approval Date: 12/05/2015).

### 3.5. Locomotor Performance

To evaluate the possible non-specific muscle relaxant effect of DEAB, mice were tested with a rotarod apparatus (Insight Ltd., Ribeirão Preto, Brazil), based on the method of Dunham and Miya (1957) [33]. Twenty-four hours before the test, male Swiss mice that were capable of remaining on the rotarod (4 cm in diameter, 6 rpm) for three periods of 60 s without falling were preselected. For the test, the animals (*n* = 8) received the vehicle (saline, 10 mL/kg, oral by gavage), DEAB (300 mg/kg, oral), or diazepam (2 mg/kg, intraperitoneal) as a positive control. The DEAB selected dose of 300 mg/kg in this test was selected based on the maximum dose already evaluated by Da Rocha et al. [5]. One hour after oral treatment with saline or DEAB and 30 min after diazepam injection, the mice were placed on the apparatus. The number of falls from the apparatus was recorded with a stopwatch for 180 s.

### 3.6. Formalin-Induced Nociception

The model that was used has been described by Hunskaar and Hole in 1987 [11], with a few modifications. The mice (*n* = 8–10) were treated with DEAB (10, 30, or 100 mg/kg, oral), vehicle (10 mL/kg, oral), morphine (2.5 mg/kg, subcutaneous) as a positive control for the neurogenic phase, or piroxicam (30 mg/kg, oral) as positive control for the inflammatory phase. One hour after oral treatments and 30 min after morphine administration, the mice received an intraplantar injection in their right hind paw of a formalin/saline solution (20 µL, 1% formaldehyde). After formalin injection, the animals were immediately placed into 20 cm glass cylinders, and the time (s) spent licking the injected paw was recorded with a chronometer as an indicator of nociception. The mice were observed during the first 5 min (neurogenic phase) and between the fifteenth and thirtieth minute (inflammatory phase).

### 3.7. Hot Plate Test

Thermal hypersensitivity after DEAB administration was evaluated with a hot plate test [34]. The animals (*n* = 8) were treated with DEAB (30 mg/kg, oral), vehicle (10 mL/kg, oral), or morphine as a positive control (5 mg/kg, subcutaneous). One hour after the oral and 30 min after the subcutaneous treatments, the mice were placed on a heated metal plate (Ugo Basile, Varese, Italy) with the temperature set at 56 ± 1 °C. The time (s) until the mouse manifested a nociceptive behavior (lifting or licking its hind-paw) was considered the latency response to the thermal stimuli. A cut-off time of 30 s was chosen to avoid tissue injury. This latency response was recorded 60, 90, and 120 min following oral treatment.

### 3.8. Involvement of Transient Receptor Potential Cation Channel Subfamily V Member 1, A Member 1, and M Member 8 (TRPV1, TRPA1, and TRPM8, Respectively) and Acid-Sensing Ion Channel (ASIC)

To evaluate the involvement of TRPV1, TRPA1, TRPM8, and ASIC channels in the antinociceptive activity of DEAB, we used specific activators of each channel. Male Swiss mice (*n* = 8) were pretreated with DEAB (30 mg/kg) or saline (10 mL/kg) orally one hour before algogenic injections. Then, the mice received 20 µL (intraplantar injection) of capsaicin (2 µmol/paw), cinnamaldehyde (40 nmol/paw), menthol (2 µmol/paw), or acidified saline (3% acetic acid, pH = 2) into the ventral surface of the right hind paw. Animals were placed individually in a glass cylinder and were observed for 6 min (TRPV1 and TRPA1) and for 20 min (TRPM8 and ASIC), according to the procedures outlined in previous publications [35,36], with modifications. The amount of time (s) spent licking the injected paw was recorded and considered indicative of nociception.

### 3.9. Involvement of Glutamatergic System

To evaluate the involvement of the glutamatergic system, the animals (*n* = 8–10) received a 20 µL glutamic acid injection (30 µmol/paw, pH = 7, intraplantar injection) in their right hind paw, one hour after DEAB (30 mg/kg) or saline (10 mL/kg) oral treatments. After the injection, the animals were observed for 15 min. The time, in seconds, that each mouse spent licking its right hind paw was used as the nociception indicator [37].

### 3.10. Involvement of the Opioid System

To assess whether the opioid system mediated the antinociceptive effect of DEAB, mice (*n* = 8–10) received naloxone, a non-selective opioid receptor antagonist (1 mg/kg, intraperitoneal), and, after 30 min, were treated with DEAB (30 mg/kg, oral), vehicle (10 mL/kg, oral), or morphine (2.5 mg/kg, subcutaneous) as a positive control. One hour after oral treatments and 30 min after morphine administration, the mice received 20 µL of a formalin/saline solution (1% formaldehyde, intraplantar injection) in their right hind paw, and were observed during the first 5 min (neurogenic phase). The time, in seconds, that each mouse spent licking its right hind paw was used as the nociception indicator [38,39].

### 3.11. Statistical Analyses

The results are expressed as means ± standard error of the mean of the parameters obtained. The parameters were analyzed using one-way ANOVA followed by Dunnett’s test to compare three or more groups, using Student’s *t*-test to compare two groups, or using two-way ANOVA followed by Bonferroni’s test to compare three groups with repeated measures. Data were analysed using the software GraphPad Prism 6.0. The minimal significance level considered was *p* < 0.05.

## 4. Conclusions

*Arrabidaea brachypoda* (DC) Bureau is a medicinal plant that is popularly used in Brazil as a natural therapeutic agent to treat pain and inflammation. This study evaluates the antinociceptive activity of the DEAB obtained from the roots of this plant. The dichloromethane fraction of *A. brachypoda*, containing a mixture of the compounds brachydin A (**1**), brachydin B (**2**), and brachydin C (**3**), alleviates acute pain in mice. Our data suggest the involvement of the TRPM8 channels and ASIC receptors, as well as the opioid system in the acute pain relief. The effective antinociceptive activity of this plant is probably related with the presence of the previously described glycoside derivatives. Possibly, after oral ingestion, these compounds will be hydrolyzed in the acid pH of the stomach, liberating the aglycones present in the dichloromethane fraction described in this study. This hypothesis is under investigation.

This study makes a significant contribution to the literature because it provides a mechanistic insight into the pain-alleviating activities of this important medicinal plant, and may explain its traditional use for local populations in the Brazilian Cerrado ecosystem.
